# Insecticide susceptibility of *Anopheles stephensi* to DDT and current insecticides in an elimination area in Iran

**DOI:** 10.1186/s13071-016-1851-4

**Published:** 2016-11-04

**Authors:** Mehdi Zare, Moussa Soleimani-Ahmadi, Sayed Hossein Davoodi, Alireza Sanei-Dehkordi

**Affiliations:** 1 Department of Occupational Health Engineering, Faculty of Health, Hormozgan University of Medical Sciences, Bandar Abbas, Iran; 2 Social Determinants in Health Promotion Research Center, Hormozgan University of Medical Sciences, Bandar Abbas, Iran; 3 Department of Medical Entomology and Vector Control, Faculty of Health, Hormozgan University of Medical Sciences, P.O. Box: 79145–3838, Bandar Abbas, Iran; 4 Department of Nutrition Research, National Nutrition and Food Technology Research Institute, Faculty of Nutrition Sciences and Food Technology, Shahid Beheshti University of Medical Sciences, Tehran, Iran

**Keywords:** Insecticide resistance, *Anopheles stephensi*, Malaria, Jask, Iran

## Abstract

**Background:**

Iran has recently initiated a malaria elimination program with emphasis on vector control strategies which are heavily reliant on indoor residual spraying and long-lasting insecticidal nets. Insecticide resistance seriously threatens the efficacy of vector control strategies. This study was conducted to determine the insecticide susceptibility of *Anopheles stephensi* to DDT and current insecticides in Jask county as an active malaria focus in southeastern Iran.

**Methods:**

In this study, the anopheline larvae were collected from different aquatic habitats in Jask county and transported to insectarium, fed with sugar and then 3-day-old adults were used for susceptibility tests. WHO insecticide susceptibility tests were performed with DDT (4 %), malathion (5 %), lambda-cyhalothrin (0.05 %), deltamethrin (0.05 %) and permethrin (0.75 %).

**Results:**

The field strain of *An. stephensi* was found resistant to DDT and lambda-cyhalothrin. The LT_50_ values for DDT and lambda-cyhalothrin in this species were 130.25, and 37.71 min, respectively. Moreover, *An. stephensi* was completely susceptible to malathion and permethrin and tolerant to deltamethrin.

**Conclusion:**

The present study results confirm the resistance of the major malaria vector, *An. stephensi*, to DDT and lambda-cyhalothrin, and tolerance to deltamethrin, which could gradually increase and spread into other malaria endemic areas. Thus, there is a need for regular monitoring of insecticide resistance in order to select suitable insecticides for vector control interventions towards malaria elimination.

## Background

Malaria is one of the foremost public health problems in Iran. Recent studies on anopheline mosquitoes in Iran have reported the presence of eight malaria vectors [1]. In the southeastern Iran six species have been incriminated as malaria vectors, i.e. *Anopheles culicifacies*, *An. dthali*, *An. fluviatilis*, *An. stephensi*, *An. superpictus* and *An. pulcherrimus*, noting that the major vector in the area is *An. stephensi* [1–3].

**Fig. 1:**
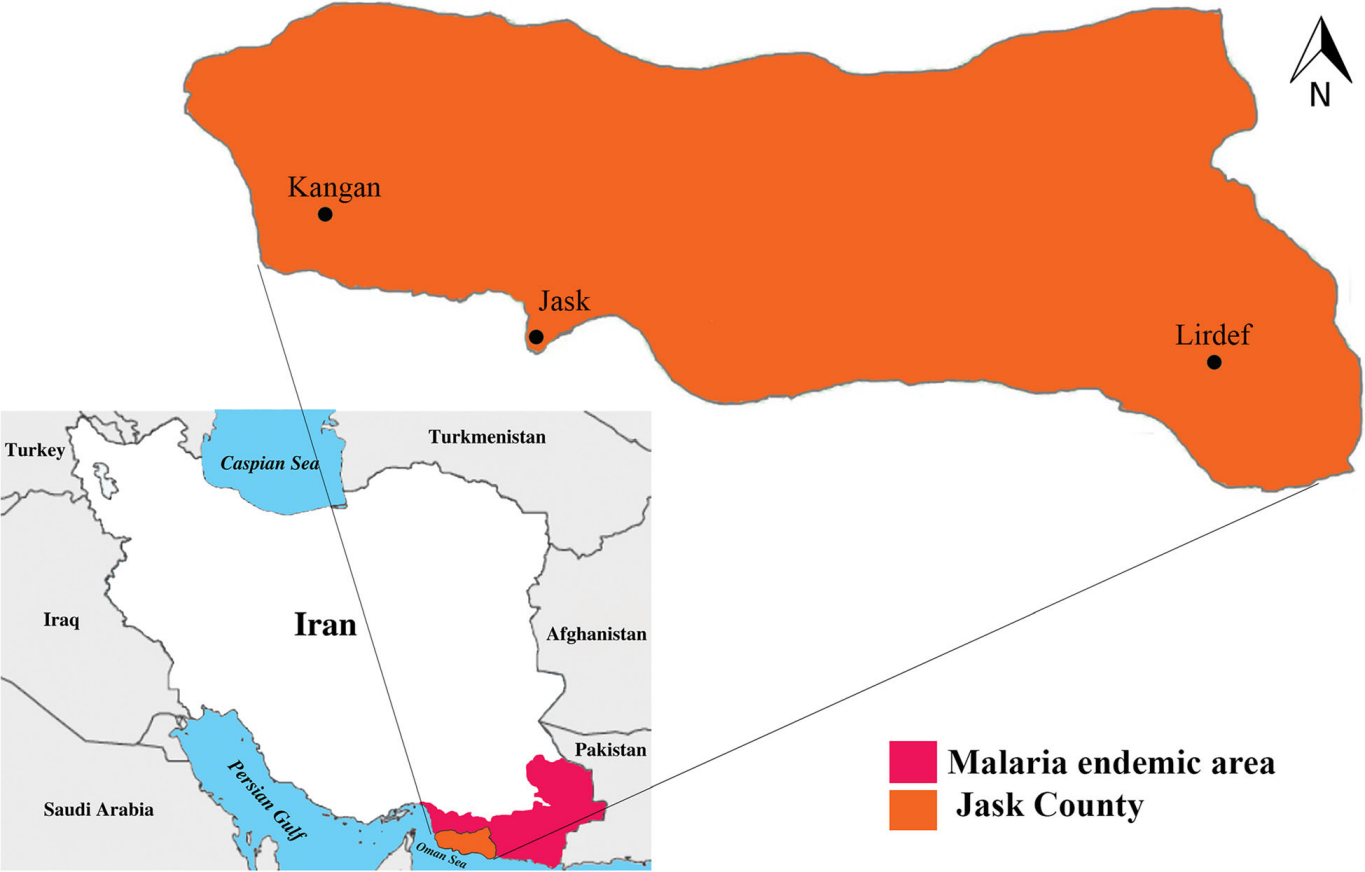
Map of Iran, highlighting the location of malaria endemic areas and study area in Jask county

The initial malaria eradication campaign was initiated in Iran in 1951 and changed to malaria control in 1985 as a result of constraints and challenges [4]. Iran has been in the current elimination phase since 2010 and the number of indigenous cases were reduced from 1850 cases in 2010 to 358 in 2014 [5]. At present, almost all regions are free of the disease, with the exception of the thinly populated southeastern tropical part of the country near the border with Pakistan (Fig. 1). This area has two seasonal peaks in spring and autumn [1, 6].

Vector control is the main component of the malaria control strategy, which aims to prevent parasite transmission through interventions targeting anopheline vectors [7]. The lack of an effective malaria vaccine and the presence or emergence of resistance to existing anti-malarial drugs further increases the importance of vector control measures [8]. Vector control for malaria is mainly composed of long-lasting insecticidal nets (LLINs) and indoor residual spraying (IRS) programmes which are highly dependent on pyrethroid insecticides [9]. These insecticides are the only class approved for use on LLINs and they are being increasingly used in IRS because of their low human toxicity, excito-repellent properties, rapid rate of knock-down and killing effects [10]. Moreover, pyrethroid insecticides are being widely used in the control of agricultural pests worldwide [11]. The extensive use of pyrethroids has increased the selection pressure on the major malaria vectors, which have inevitably developed resistance [12]. In Iran, resistance of the main malaria vector *An. stephensi* to DDT and malathion has been reported from the southeast areas, where the first indication of this species resistance to pyrethroid insecticides was reported in 2012 [13, 14].

Successful implementation of IRS and LLINs as the main malaria vector control strategies requires sound and up-to-date information on susceptibility of the anopheline species involved in malaria transmission to available insecticide compounds. Therefore this study was conducted to determine the susceptibility of natural population of *An. stephensi* as the major malaria vector to DDT and current insecticides in Jask county which is an active malaria focus under elimination programme in Iran. The results of this study will provide information that would help in planning and implementing an effective program for vectors control during elimination phase by the National Malaria Control Program.

## Methods

### Study area

**Fig. 2:**
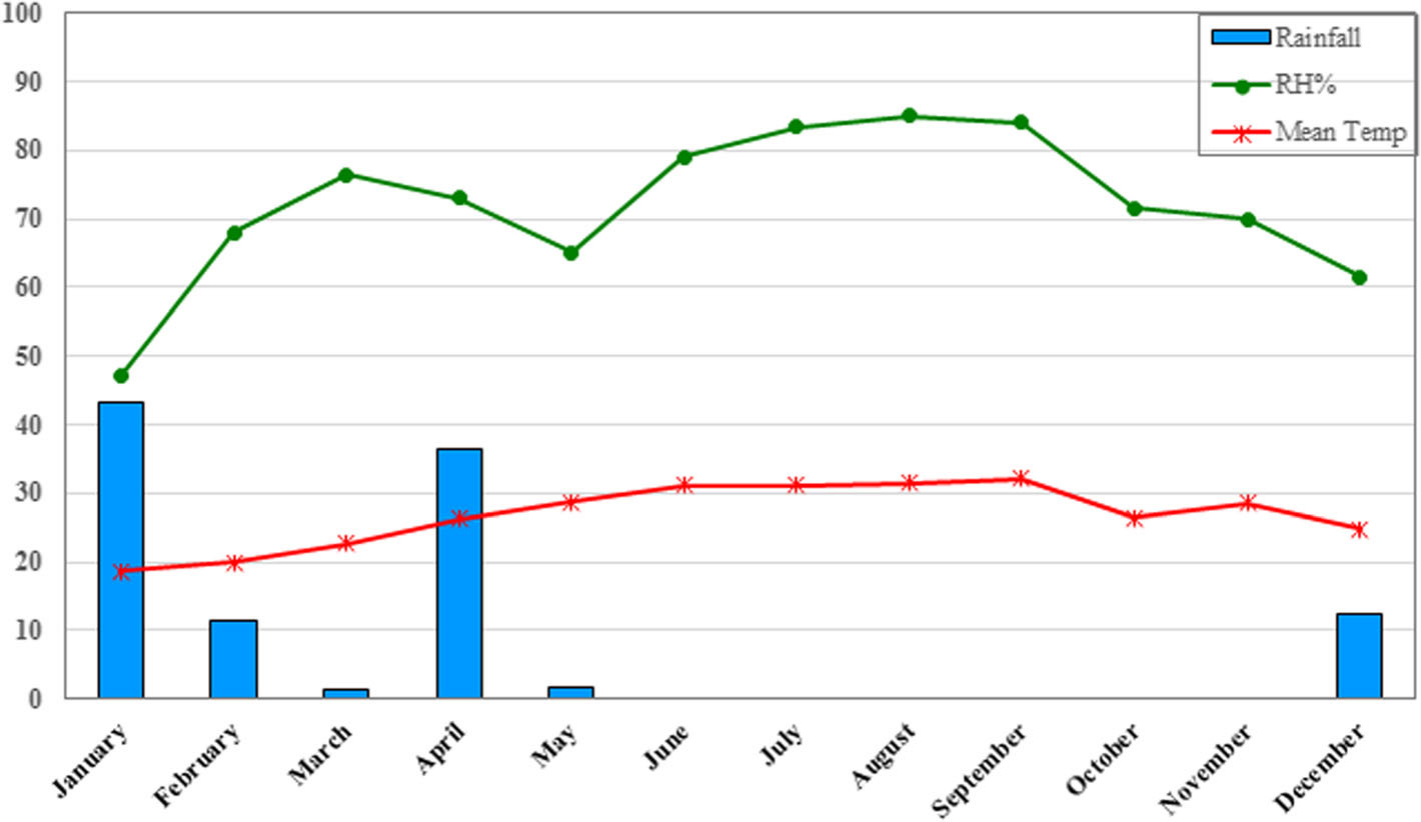
Average of meteorological parameters during 2013–2014 in Jask county, southeastern Iran

This study was carried out in Jask County in the Hormozgan province, southeastern Iran. The county has an area of 11,141 km^2^ and is located between latitudes 25°23'–26°13'N and longitudes 57°10'–59°16'E, with an approximately 78,700 population in 2015 (Fig. 1). The Jask County has a dry and warm climate with hot summers and temperate winters and mean annual temperature of 26.8 °C ranging from 18.7 °C to 32.2 °C. The rainy season occurs between December and May with an annual average of 106.7 mm. The averages of minimum and maximum relative humidity are 47 % in January and 85 % in August, respectively (Fig. 2). More than 90 % of the area is located in plain/coastal area with an altitude of less than 400 m and the rest is mountainous with an altitude of more than 400 m. This county is an agricultural region irrigated by rivers which are the main breeding sites for anopheline mosquitoes. Agriculture, livestock herding, fishing and trading are the main occupations in the study area.

Jask conty is one of the most active malaria foci in the southeast of Iran and malaria cases are reported in this area year-round with peaks after the two annual rainy seasons (April-June and September-December) [15].

### Mosquito collection and rearing

Since *An. stephensi* is the main malaria vector in the south of Iran, insecticide tests were performed on this species during spring and fall 2014. Anopheline larvae were collected at different times during the mosquito breeding season from December 2013 to August 2014 from different habitats such as river streams, water puddles, and stagnant water areas where the probability to find larvae is high. The larvae samples were transferred to the laboratory and reared to adult stage under standard conditions at 25–29 °C, 12:12 light: dark photocycle, and 50–70 % relative humidity in the insectary of Bandar-Abbas Health School. Larvae were reared in plastic trays (35 × 25 × 10 cm) containing 2.5 l of deionized water at a density of approximately 100 larvae/l and fed with fish food. Pupae were transferred to screened cages and emerged adults were fed with 10 % sugar solution. The F1 progeny when 2–3-day-old sugar-fed females were used for the tests. Mosquitoes were identified morphologically using standard keys used for the identification of anopheles of Iran [16].

### Insecticide susceptibility tests

Insecticide susceptibility tests were carried out using the WHO susceptibility test kits and standard procedures with four replicates of 25 adult female mosquitoes per test tube [17]. Mosquitoes were exposed to papers impregnated with the WHO-recommended discriminating concentrations of six insecticides including DDT (4 %), malathion (5 %), lambda-cyhalothrin (0.05 %), deltamethrin (0.05 %) and permethrin (0.75 %) (supplied by WHO). Mineral oil, olive oil and silicon oil impregnated papers were respectively used for organochlorine, organophosphate and pyrethroid insecticides as controls [17].

The exposure time for each insecticide was 1 h. The mortality rate was calculated after a 24 h of recovery period. For those insecticides that the observed mortalities in the test were below 90 %, mortality rates in various time intervals were also calculated and then regression lines were plotted. KDT_50_ and KDT_90_ values were calculated using Probit analysis. Error bars for each mortality rate were calculated based on statistical method at α = 5 %.

### Statistical analysis

Mortality was determined by counting the dead and alive mosquitoes at the end of the 24 h of recovery period. The mortality then was corrected by applying Abbott’s formula when control mortality was recorded between 5 and 20 %, while tests with > 20 % control mortality were discarded and repeated [18]. Resistance was defined according to WHO guidelines which suggests that 98–100 % mortality indicates susceptibility, 90–97 % indicates the possibility of resistance that needs to be confirmed, and < 90 % indicates resistance [17].

## Results

**Fig. 3:**
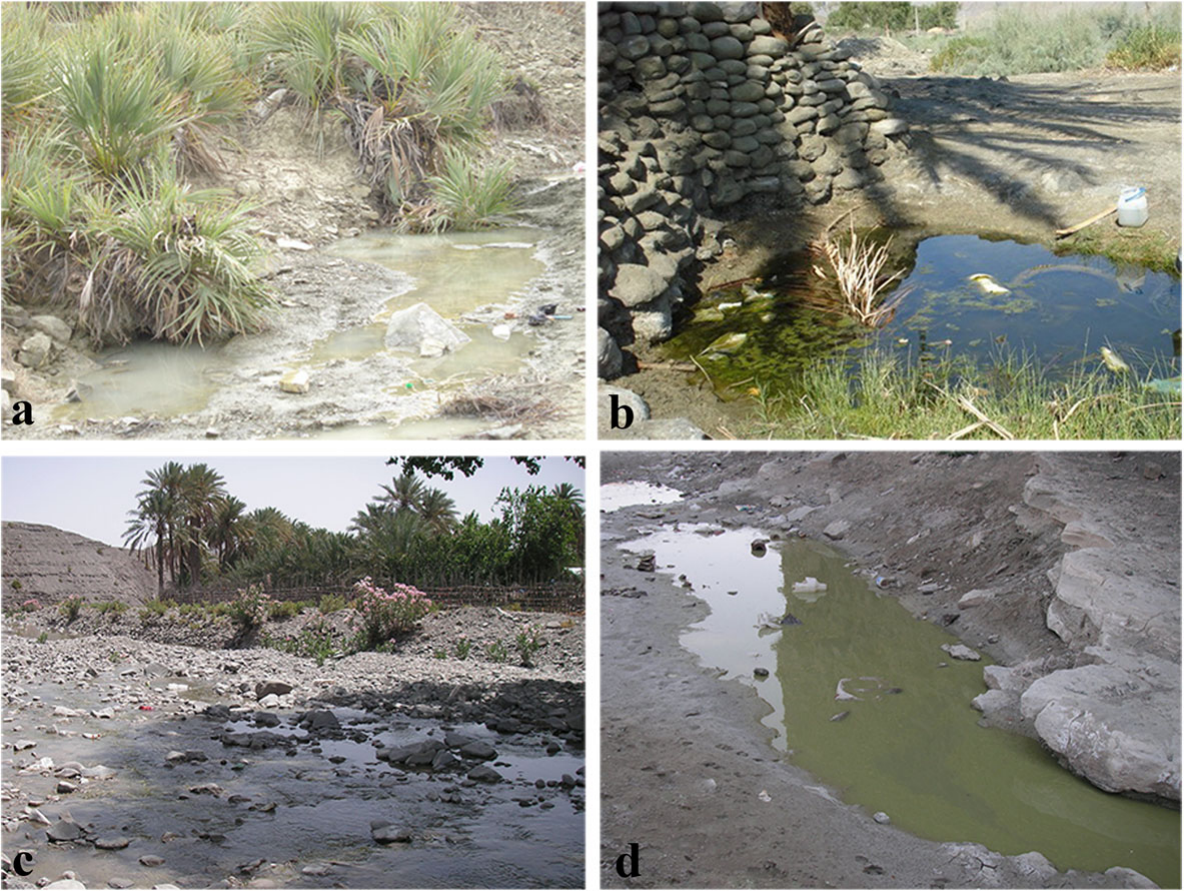
Typical potential anopheline larval habitats in Jack county, southeastern Iran. **a**, **b** Water leakage. **c** Riverbed. **d** River edge

A total of 1500 female mosquitoes, reared from larvae collected in different habitats (Fig. 3), were exposed to insecticides belonging to the three WHO approved classes.

**Fig. 4:**
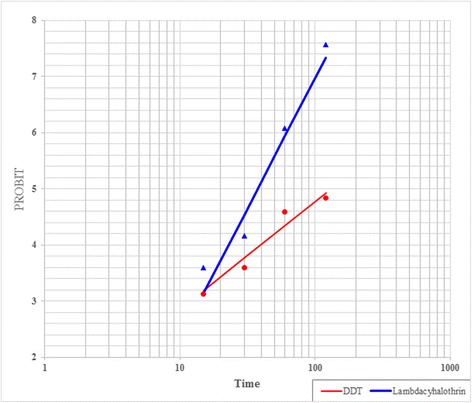
Regression lines of *An. stephensi* exposed to DDT (4 %) and Lambda-cyhalothrin (0.05 %) in southeastern Iran

The results of susceptibility tests using a threshold of 90 % mortality for resistance confirmation as set by WHO criteria indicated that the natural population of *An. stephensi* is resistant to DDT. The mortality rate of *An. stephensi* to DDT was 66 % which indicates a high level of resistance to DDT. The predicted LT_50_ and LT_90_ values for this species were 130.25 and 597.35 min, respectively. Mortality rate of *An. stephensi* mosquitoes that were exposed for 60 min to 0.05 % lambda-cyhalothrin was 86 %, indicating the resistance of this species to lambda-cyhalothrin. The predicted LT_50_ and LT_90_ values of lambda-cyhalothrin against *An. stephensi* were 37.71 and 71.12 min, respectively. The results of the study on the efficacy of DDT and lambda-cyhalothrin and regression line parameters are presented in Table 1. Regression line showed a linear relationship between mortality rate and exposure time (Fig. 4).

**Table 1:** Probit regression line parameters of *An. stephensi* exposed to DDT (4 %) and Lambda-cyhalothrin (0.05 %) in southeastern Iran

Insecticides	A	B ± SE	LT_50_ 95 % C.I. (min)	LT_90_ 95 % C.I. (min)	*χ* ^2^ (*df*)	*P*-value
DDT (4 %)	-4.097	1.94 ± 0.246	130.25	597.35	5.23 (3)	> 0.05
Lambda-cyhalothrin (0.05 %)	-7.33	4.65 ± 1.096	37.71	71.12	16.44 (2)	< 0.05

*Abbreviations*: A, intercept; B, slope; SE, standard error; LT_50_, 95 % CI, lethal time causing 50 % mortality and its 95 % confidence interval; LT_90_, 95 % CI, lethal time causing 90 % mortality and its 95 % confidence interval; *χ* ^2^, heterogeneity about the regression line; df, degrees of freedom

The results also showed that *An. stephensi* was susceptible to malathion and permethrin, and tolerant to deltamethrin. The results of insecticide susceptibility tests carried out on *An. stephensi* mosquitoes are shown in Table 2.

**Table 2:** Insecticide susceptibility status of natural population of *An. stephensi* in southeastern Iran

Insecticide (%)	Exposure time (min)	Treatment			Control			Resistance status
		No. exposed (Replicates)	No. dead	Mortality rate (%)	No. exposed (Replicates)	No. dead	Mortality rate (%)	
DDT (4 %)	15	100 (4)	3	3	50 (2)	0	0	R
	30	100 (4)	8	8	50 (2)	0	0	
	60	100 (4)	34	34	50 (2)	0	0	
	120	100 (4)	52	52	50 (2)	4	4	
Lambda-cyhalothrin (0.05 %)	15	100 (4)	8	8	50 (2)	0	0	R
	30	100 (4)	20	20	50 (2)	0	0	
	60	100 (4)	86	86	50 (2)	0	0	
	120	100 (4)	100	100	50 (2)	3	3	
Deltamethrin (0.05 %)	60	100 (4)	95	95	50 (2)	0	0	T
Permethrin (0.75 %)	60	100 (4)	100	100	50 (2)	5	5	S
Malathion (5 %)	60	100 (4)	100	100	50 (2)	2	2	S

*Abbreviations*: *R* Resistance, *T* Tolerance, *S* Susceptible

## Discussion

The Iran national malaria control programme has implemented indoor residual spraying with lambda-cyhalothrin and deltamethrin insecticides, distribution of long-lasting insecticidal nets and application of larvicides as the main malaria vector control strategies [1, 14, 19]. Development of insecticide resistance in malaria vectors is one of the serious limitations of effective vector control strategies that rely on chemical insecticides. It has been reported that rapid emergence and geographical spread of insecticide resistance among malaria vectors has threatened the intervention programmes in many endemic Afro-Asian countries [5, 9, 13]. Therefore, regular monitoring of insecticide susceptibility among endemic malaria vectors is an essential component for the development of an effective vector management plan.

Since *An. stephensi* is the most prevalent endophilic species and has been confirmed as the main malaria vector in the south of Iran [1], this study conducted to evaluate insecticide susceptibility status of this species against DDT and current insecticides which have been used in malaria control programs. In this study, *An. stephensi* was found resistant to DDT. This finding is in agreement with previous studies conducted in the malarious areas of southern Iran [20]. In Iran, insecticide resistance of this species to DDT was reported for the first time in 1957. Recent studies in Chabahar and Bashagard, the neighbouring counties of the study area, have shown that *An. stephensi* is resistant to DDT [13, 20]. Moreover, susceptibility tests in neighbouring countries including Afghanistan, Pakistan, Iraq, Oman, United Arab Emirates, and Saudi Arabia have shown resistance of *An. stephensi* to DDT [21]. *Anopheles stephensi* is sufficiently endophilic and endophagic to come into contact with insecticide residues in houses and therefore insecticide resistance in this species can be explained by the widespread use of DDT house spraying in different areas of Iran during the malaria eradication campaign in 1950s [13, 20]. Although there is no report of cross-resistance between DDT and pyrethroids, it seems widespread use of pesticides in agricultural activities and increased coverage of LLINs as a malaria vector control measure can potentially lead to such cross-resistance.

The results of this study indicated the susceptibility of *An. stephensi* to malathion. Although resistance of this species to malathion had been previously reported from the south of Iran in 1976, reduction of resistance of *An. stephensi* to malathion has been well-notified in the recent years by various researchers [20].

The results of this study also revealed the resistance of *An. stephensi* to lambda-cyhalothrin, tolerance to deltamethrin, and sensitivity to permethrin. Approximately in all previous conducted studies on *An. stephensi* in Iran, susceptibility to pyrethroids has been reported, but in 2012 the first indication of this species resistance to pyrethroid insecticides was reported from the southeastern parts of the country [13]. Moreover, the results of a recent study in Chabahar county in Sistan va Baluchestan Province showed that this species is resistant to lambda-cyhalothrin and tolerant to deltamethrin with mortality rates of 89 and 96 %, respectively [22]. Similar findings have been reported from malaria endemic areas in Afghanistan [23]. Synthetic pyrethroids including deltamethrin and lambda-cyhalothrin are being widely used in various public health programmes to control mosquitoes in many countries. However, in the recent years the efficacy of these insecticides against potential malaria vectors has been found to be reduced in endemic areas [24, 25]. In this regard, resistance of the malaria vectors to pyrethroids have been reported from some Afrotropical regions including Mozambique, Uganda, southern Africa, Benin and Ghana [26].

Resistance of *An. stephensi* against lambda-cyhalothrin in this study may be explained by massive use of these insecticides in IRS during past years and its tolerance to deltamethrin which was found in this study may be due to large scale distribution of deltamethrin-impregnated LLINs and its use in house spraying applications during recent years. Moreover it seems that repeated use of pyrethroid insecticides as agricultural pesticides has resulted in a selection pressure on mosquito populations in the study area leading to the development of insecticide resistance. Similar trends have been reported from Middle East different regions, Indian subcontinent, and many African malaria endemic countries [23, 24, 27]. The use of pyrethroids as pesticides in agriculture and bed-net treatment has been recognized as factors responsible for the natural selection of resistant mosquitoes in sub-Saharan Africa [26, 28].

The results of this study showed the resistance of *An. stephensi* as the main malaria vector to pyrethroid insecticides in the southeast of Iran. The emergence and rapid spread of insecticide resistance in *Anopheles* population may impair the effectiveness of malaria vector control measures which are based on the use of LLINs and IRS and therefore threaten the sustainability of the current strategies for malaria elimination [26, 29, 30]. Therefore, to implement a successful malaria control program it is vital to take into account the implications of insecticide resistance to pyrethroids. In this regard, utilization of biochemical and molecular assays are recommended to understand the mechanisms of pyrethroid resistance in *An. stephensi* to implement targeted vector control interventions and predict the origin and likely impact of the resistance.

## Conclusion

This study confirms the resistance of the major malaria vector, *An. stephensi*, to DDT and lambda-cyhalothrin and reduced susceptibility to deltamethrin which could gradually increase and spread into other malaria endemic areas. Since the resistance level of *An. stephensi* against insecticides may decrease the efficacy of LLINs and IRS, there is a need for regular monitoring of current insecticides resistance in order to select suitable insecticides for vector control interventions towards malaria elimination. In this regard, resistance management strategies including rotations of insecticides, use of interventions in combination, mosaic spraying, and use of mixtures should be considered to control insecticide resistance in the *Anopheles* population in malaria endemic areas to improve malaria elimination programs.

## Abbreviations

IRS: Indoor residual spraying
LLINs: Long-lasting insecticidal nets
